# Acid-activated chitosan-modified Iranian bentonite for adsorption of Cu(II) and Zn(II) from water; A green and sustainable water treatment approach

**DOI:** 10.1371/journal.pone.0344207

**Published:** 2026-07-30

**Authors:** Sam Daftari, Mojgan Salavati, Manoochehr Mortazavi Chamchali

**Affiliations:** Department of Geology, La.C., Islamic Azad University, Lahijan, Iran; University of South Africa, SOUTH AFRICA

## Abstract

This study evaluates the adsorption performance of an Iranian bentonite modified through two routes: activation with HNO_3_ followed by calcination at 500 °C yielding NWF adsorbent and activation with HCl followed by chitosan intercalation to obtain HWF adsorbent. Batch experiments assessed Cu^2+^ and Zn^2+^ removal from aqueous solutions under varying pH, contact time (5–45 min), sorbent dosage (10–150 mg), initial concentration (20–200 mg/L), and temperatures (20–45°C). Atomic Absorption Spectroscopy was used to evaluate heavy metal concentrations. Nonlinear isotherm fitting showed that the Langmuir model described the data well with high mass normalized capacities under the low-dose condition. Freundlich fits indicated favorable adsorption on heterogeneous surfaces. Kinetics followed the pseudo-second-order model (R² = 0.996–0.998), could be described by chemisorption and in case of NWF, faster uptake (higher k_2_) achieved. Optimal adsorption performance occurred at a pH of approximately 6–7, achieving equilibrium capacities (*q*_*e*_) as high as 99.0 mg/g and blank tests at pH ≥ 8 minimized contributions from metal hydroxide precipitation. Thermodynamic analysis has been carried out to confirm spontaneous and endothermic adsorption in temptatures ranged from 20–45°C. X-ray Diffraction, Fourier Transform Infrared Spectroscopy and Scanning Electron Microscopy corroborated successful modification and enhanced site accessibility. The results of this study showed that acid-activated and chitosan-modified bentonites are low-cost, effective, and sustainable sorbents for Cu^2+^ and Zn^2+^ removal from water, with practical relevance to wastewater treatment.

## 1. Introduction

Metals such as copper and zinc rank among the most hazardous pollutants in aquatic ecosystems when they are discharged from industries like electroplating, metal finishing, mining, and chemical processing. Their high toxicity, tendency to bioaccumulate, and resistance to degradation pose severe long-term risks to ecosystems and human health [1]. Among these pollutants, copper (Cu²⁺) and zinc (Zn²⁺) are particularly important due to their widespread industrial use, environmental persistence, and toxicity at elevated concentrations. Even at trace levels, Cu²⁺ and Zn² ⁺ can disrupt aquatic life and accumulate in biological tissues, leading to neurotoxicity, gastrointestinal disorders, and potential carcinogenic effects [1,2]. Conventional remediation methods—including chemical precipitation, membrane filtration, ion exchange, and electrochemical processes—often require high energy inputs and generate secondary wastes; moreover, their efficiency declines at low metal concentrations [2]. In contrast, surface adsorption is widely regarded as an efficient, flexible, and cost-effective alternative particularly when employing naturally occurring or low-cost sorbents such as clay minerals [3]. Indeed, the field of water remediation has seen a surge in the development of advanced materials beyond traditional sorbents. These include sophisticated polymer-based nanocomposite membranes designed for selective heavy metal removal [4], and advanced functional materials like metal-organic frameworks (MOFs) which are heavily researched for applications such as photocatalytic CO_2_ reduction [5]. Other approaches employ hydrogel composites [6] or advanced electrochemical methods for the sensing and degradation of persistent organic pollutants [7]. While these high-performance materials demonstrate significant capabilities, their application can often be limited by high cost, complex synthesis, and challenges in scaling. Therefore, enhancing the performance of naturally abundant and low-cost materials such as clay minerals, remains a critical and highly practical research avenue [3,8].

Bentonite, a smectite-rich layered aluminosilicate clay, is among the most widely available and economically viable adsorbents. Its high surface area, cation exchange capacity, structural flexibility, and swelling behavior make it a promising environmentally friendly sorbent [9]. In most cases, non-modified bentonite needs surface modification to achieve optimal performance under realistic conditions. To enhance adsorption on this material, several modification strategies like acid activation, polymer functionalization, calcination, and nanoparticle deposition have been explored. Acid treatments with nitric or hydrochloric acid remove impurities, expose new active sites, and improve uptake [10]. Chitosan, a biopolymer which is rich in amine and hydroxyl groups, enhances metal binding via chelation and hydrogen bonding [11,12]. Recent reviews highlight clay minerals, including modified bentonites, as strong candidates for heavy metal removal from water [3,8].

In this study, we used bentonite as the base material. The potential of bentonite for heavy metal removal has been demonstrated in prior Iranian studies. For example, Hosseini Noveh et al. reported the adsorption of Pb, Cu, and Ni from industrial effluents using Iranian bentonite, underscoring the importance of reducing heavy metals in wastewater [13]. Other domestic studies have also confirmed the effectiveness of bentonite in single- and multi-component systems for various metals [14,15]. To improve the bentonite’s adsorption capacity for Cu²⁺ and Zn² ⁺ , two chemical modifications were applied. The first route involved treatment with 2M nitric acid followed by calcination at 500 °C which yielded NWF in a process that increases surface roughness, pore accessibility, and reactive sites through partial delamination. The second route comprised 2 M hydrochloric acid activation followed by chitosan intercalation to produce HWF, which introduces amine and hydroxyl functional groups capable of chelating metal ions. The adsorption performance of NWF and HWF was evaluated and compared under varying operational conditions (pH, contact time, initial metal concentration, sorbent dosage, and temperature). The results demonstrate the promise of modified Iranian bentonite as a sustainable and scalable adsorbent for water treatment, with direct relevance to industrial wastewater applications in mining-affected regions.

## 2. Materials and methods

### 2.1. Materials

Bentonite was obtained from the Suravajin Aghigh mine in southwestern Boein Zahra (Iran), located within the Urmia–Dokhtar magmatic arc characterized by volcanic and pyroclastic lithologies and extensive Eocene hydrothermal alteration of tuffaceous rocks. Mineralogical investigations by the Geological Survey of Iran (XRD/FTIR) indicate a predominantly illitic clay (≈95% illite) with minor montmorillonite (≈5%) and accessory quartz, kaolinite, chlorite, feldspar, dolomite, and calcite; the average stoichiometric formula can be represented as [16]:


$$\begin{array}{c} ({\text{Na}}_{\text{0}.\text{1}\text{2}}{\text{K}}_{\text{0}.\text{6}\text{2}}{\text{Ca}}_{\text{0}.\text{0}\text{6}}){\text{Si}}_{\text{4}.\text{1}}({\text{Al}}_{\text{1}.\text{3}}{\text{Fe}}_{\text{0}.\text{2}\text{3}}{\text{Mg}}_{\text{0}.\text{0}\text{7}}){\text{O}}_{\text{1}\text{0}}{\left(\text{OH}\right)}_{\text{2}}. \end{array}$$

To ensure a representative and high-purity starting material, a grab-sampling method was employed at five distinct locations within the quarry. Each of these primary samples was screened using XRD and FTIR. The sample exhibiting the highest illite content and the fewest accessory mineral impurities (e.g., quartz, calcite) was selected for all subsequent modification experiments. The selected samples were crushed, sieved to 100 mesh, and thoroughly washed with double-distilled water to remove soluble impurities, and oven-dried at 105 °C for 24h. All chemicals used in this study were of analytical or ACS grade. Nitric acid (HNO₃, 70%) and hydrochloric acid (HCl, 37%) were purchased from Merck (Germany), sodium hydroxide (NaOH, ≥ 98%) and acetic acid (CH₃COOH, ≥ 99.7%) were obtained from Sigma-Aldrich (USA). Copper(II) nitrate trihydrate (Cu(NO₃)₂·3H₂O) and zinc sulfate heptahydrate (ZnSO₄·7H₂O), both with ≥99% purity, were supplied by Sigma-Aldrich as metal ion sources for stock solution preparation. Chitosan powder (high molecular weight, 1000–2000 cps, CAS: 9012-76-4) was obtained from Tahurkaban Bahador Pars Co. (Shiraz, Iran). According to the product datasheet, the chitosan had a degree of deacetylation of 92.0% and an average molecular weight of 1,500,000 Da.

### 2.2. Preparation of modified adsorbents

For the HNO_3_-activated sample (NWF), 50 g of bentonite was treated with 500 mL of 2 M HNO_3_ at 80 °C for 2 hours, washed repeatedly with deionized water until neutral filtrate pH, dried at 105 °C, and calcined at 500 °C for 4 hours in a muffle furnace to promote partial delamination, increased pore accessibility, and removal of residual organics. For the HCl/chitosan-modified sample (HWF), another 50 g portion was treated with 2 M HCl under similar thermal conditions, washed and dried, then dispersed in 1% (w/v) chitosan (prepared in 1% acetic acid) and stirred for 24 hours at room temperature; the composite was filtered, washed, and dried at 70 °C. These procedures follow established protocols for acid activation and biopolymer functionalization of bentonites, aiming to increase surface accessibility and introduce amine/hydroxyl groups that facilitate complexation with metal cations. The specific acid activation parameters used here (e.g., 2 M, 80 °C, 2 h) are directly based on established protocols for bentonite modification [17,18], while the chitosan functionalization follows similar biopolymer composite methods [8,11,12,19].

### 2.3. Material characterization

Structural and chemical changes induced by modification were examined by X-ray diffraction (XRD), Fourier transform infrared spectroscopy (FTIR), and scanning electron microscopy (SEM). XRD patterns were recorded on a Philips X’Pert PRO diffractometer using Cu Kα radiation (λ = 1.5406 Å) over 2θ = 5–80° with 0.02° step size. FTIR spectra were obtained on a Bruker Tensor II (4000–400 cm^−1^) using KBr pellets to probe changes in clay backbone (Si–O, Al–OH) and organic components, such as amide functional groups and C–H bonds [13,20]. SEM imaging (TESCAN Mira3, 500 × –20,000×) was used to assess particle morphology, surface roughness, and apparent porosity after activation and chitosan intercalation.

### 2.4. Batch adsorption experiments

In 100 mL Erlenmeyer flasks containing 50 mL of Cu^2+^ or Zn^2+^ solution, batch tests were performed under controlled pH, contact time, initial concentration, adsorbent dosage, and temperature. The pH was adjusted between 2 and 11 using 0.1 M HCl or NaOH; to minimize contributions from hydroxide precipitation at alkaline pH, blank tests (without adsorbent) were conducted and considered in data interpretation [15,19]. All experiments were run over 5–45 min; equilibrium isotherms were obtained by varying the initial concentration (C_0_ = 20–200 mg/L) at fixed volume and dose. The effects of adsorbent dosage were evaluated for 25–150 mg adsorbent and thermodynamic measurements were carried out at 20, 30, and 45 °C. After equilibration, suspensions were centrifuged at 4000 rpm for 10 min (Centurion 1020 D.E.), and supernatants were analyzed by atomic absorption spectroscopy (NovAA 400p, Analytik Jena). Unless otherwise stated, the baseline conditions were V = 0.1 L, m = 20 mg, and C_0_ = 20 mg/L. All batch experiments were performed in triplicate, and the average values are reported. Error bars in figures represent the standard deviation of the measurements.

### 2.5. Data analysis and modeling

The adsorption capacitiy at equilibrium (*q*_*e*_) were computed by: *q*_*e*_ *= (C*_*0*_ *− C*_*e*_*)V/ m* and *q*_*t*_= *(C*_*0*_ *-C*_*t*_*) V/m*, where *C*_*0*_*,C*_*e*_ and *C*_*t*_ (mg/L) are the concentrations in initial, equilibrium, and in time-*t*; *V* (L) is the solution volume; *m* (g) is the sorbent mass; *q*_*e*_ and *q*_*t*_ are expressed in mg/g. Equilibrium adsorption data were fitted using the nonlinear Langmuir model and linearized Freundlich model. To provide a more robust statistical comparison, the goodness-of-fit was evaluated using the adjusted coefficient of determination *R*^*2*^_*adj*_, calculated based on the number of data points (n = 5) and model parameters (p = 2). Kinetic data were analyzed with the first-order and pseudo-second-order models to elucidate rate control and mechanisms [14,21,22]. Thermodynamic parameters were derived from distribution coefficients using Van’t Hoff equation to determine ΔH°, ΔS°, and ΔG° over the temprature range of 20–45 °C [23].

## 3. Results and discussion

### 3.1. Material characterization

**Fig 1:**
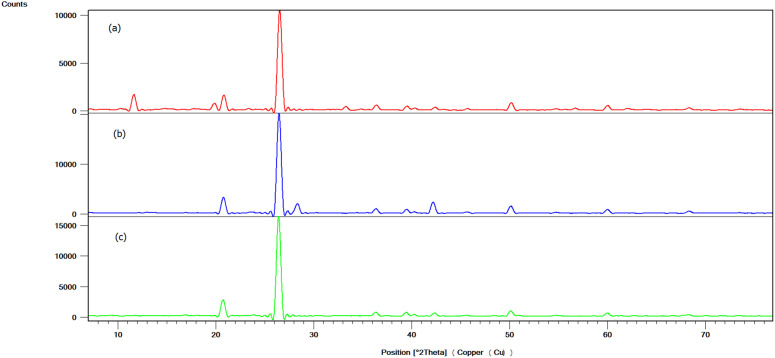
XRD patterns of (a) Suravajin bentonite, (b) NWF, and (c) HWF modified bentonites (Cu Kα, 2θ = 5–80°, step 0.02°).

XRD patterns of the Suravajin bentonite confirmed a predominantly illitic clay with a minor smectitic (montmorillonite) fraction (~5%), consistent with prior local reports on Boein Zahra clays [16] (Fig 1a). Acid activation produced clear peak broadening and subtle 2θ shifts in NWF (Fig 1b). This phenomenon is a well-established indicator of structural modification in acid-treated clays, corresponding to a partial loss of crystallinity, disruption of the layer stacking, and decomposition of the clay structure, which in turn increases interlayer/interparticle accessibility [18,24,25]. In HWF, additional small shifts were observed after chitosan intercalation, consistent with insertion of organic moieties into accessible interparticle domains (Fig 1c).

**Fig 2:**
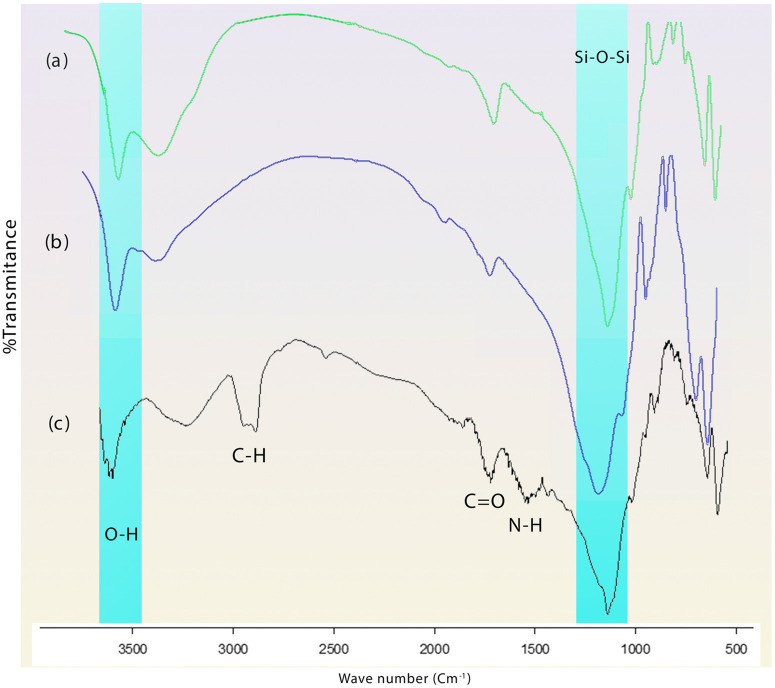
Combined FTIR spectra of (a) Suravajin bentonite, (b) NWF, and (c) HWF.

FTIR spectra were used to confirm the structural and chemical modifications. All samples exhibited the characteristic bands for the bentonite clay backbone: a broad –OH stretching vibration at approximately 3410–3620 cm ⁻ ¹ (attributed to structural hydroxyl groups and adsorbed water) and a strong, dominant Si–O–Si stretching vibration at ~1030–1050 cm ⁻ ¹ [26,27] (Fig 2a,b).

Following modification with chitosan (HWF sample), several new characteristic peaks appeared which confirm successful biopolymer incorporation (Fig 2c). These include: (i) two weak bands near ~2920 cm ⁻ ¹ and ~2850 cm ⁻ ¹, corresponding to the aliphatic C–H stretching of the chitosan polymer chain, and (ii) two critical amide-related bands. The peak at ~1650 cm ⁻ ¹ is assigned to the Amide I (C = O stretching) vibration, and the peak at ~1550 cm ⁻ ¹ corresponds to the Amide II (N–H bending) vibration [26–28]. The presence of these specific amide and aliphatic bands provides definitive evidence of chitosan functionalization on the clay surface or within its structure [27].

**Fig 3:**
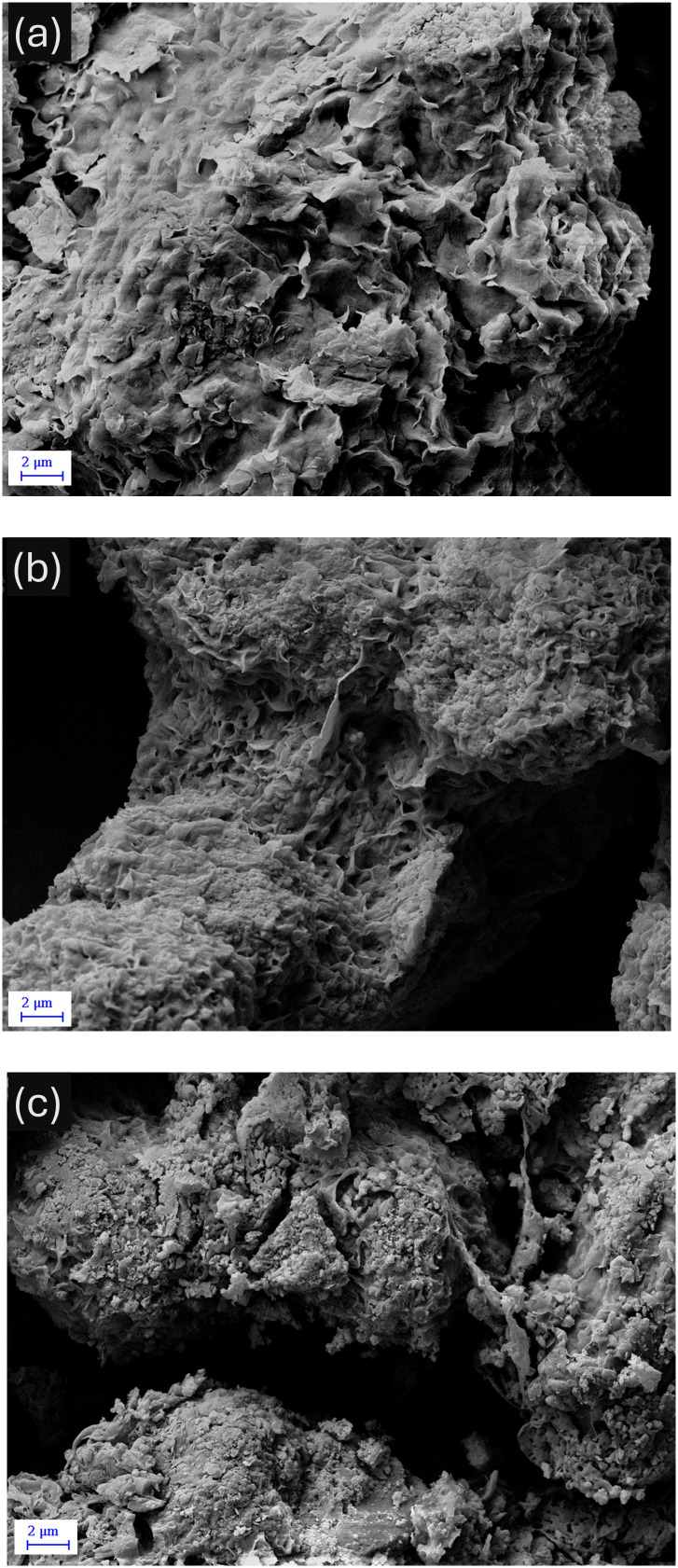
SEM micrographs of (a) Suravajin bentonite, (b) NWF and (c) HWF.

SEM micrographs revealed distinct morphological changes upon modification. The Suravajin bentonite (Fig 3a) displayed compact, plate-like aggregates typical of layered clay. Following HNO_3_ activation and calcination (NWF, Fig 3b), the surface became rougher with visible sub-micron pores and microcracks. The HCl/chitosan-modified sample (HWF, Fig 3c) exhibited a finer, more uniform surface texture with small granular features, consistent with the formation of a thin polymeric coating and rearrangement of the clay platelets [8,11,12,19].

### 3.2. Effect of pH on adsorption performance

pH critically controls adsorbent surface charge, metal-ion speciation, and the operative adsorption mechanism in solutions. Batch adsorption experiments were conducted over a pH range of 3–11 for Cu²⁺ and Zn² ⁺ using NWF (HNO₃-modified) and HWF (HCl-modified, chitosan-intercalated) adsorbents. The tests were performed at an initial concentrationat C_0_ = 20 mg/L (100 mL soloution), adsorbent mass m = 0.020 g, and temprature 25 °C. The adsorption capacity at equilibrium (*q*_*e*_) was calculated by Equation 1:


$$\begin{array}{c} q_{e}=\frac{\left(C_{\text{0}}-C_{e}\right)\times V}{m} \end{array}$$ (1)

with *C* in mg/L, *V* = 0.10 L, *m* = 0.020 g; thus *q*_*e*_ is reported in mg/g.

**Fig 4:**
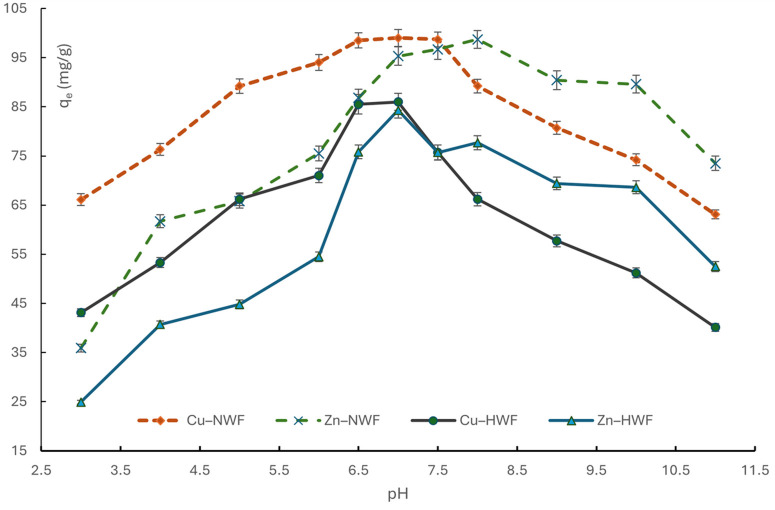
Effect of pH on equilibrium adsorption capacity (q_e_) for Cu²⁺ and Zn²⁺ on NWF and HWF (C_0_ = 20 mg/L; V = 0.10 L; m = 0.020 g; 25 °C).

As shown in Table 1 and Fig 4, q_e_ increases with pH from acidic values up to pH around 6.5–7, where maximum adsorption capacities are observed, followed by a gradual decline at higher pH values.This behavior reflects the balance of surface protonation/deprotonation and metal speciation. At acidic conditions, H^+^ ions strongly compete with metal cations for binding sites which result smaller q_e_. As pH increases, deprotonation yields more negatively charged sites, enhancing electrostatic attraction and complexation [15,29–31]. By increasing pH further and in pH ≥ 8, partial precipitation of Cu(OH)₂ and Zn(OH)₂ can contribute to an apparent plateau or decrease in q_e_. Specifically, Cu^2+^ adsorption on NWF reaches to the maximum at pH = 7, then gradually declines. Adsorption of Zn^2+^ on NWF shows a continuous increase up to q_e_ = 98.7 mg/g at pH = 8 before decreasing at higher pH values. In case of HWF, adsorption of Cu^2+^ and Zn^2+^ exhibits a smoother trend which is moderate around pH = 7, attributed to the stabilizing or buffering role of chitosan, which broadens the effective pH in adsorption process.

**Table 1:** Effect of pH on equilibrium capacity (q_e_) for Cu²⁺ and Zn²⁺ on NWF and HWF.

	q_e_ (NWF)		q_e_ (HWF)	
**pH**	**Cu** ^**2+**^	**Zn** ^**2+**^	**Cu** ^**2+**^	**Zn** ^**2+**^
**3**	66.1	35.9	43.1	24.9
**4**	76.3	61.7	53.3	40.7
**5**	89.2	65.8	66.2	44.8
**6**	94.0	75.5	71.0	54.5
**6.5**	98.5	86.8	85.5	75.8
**7**	99.0	95.3	86.0	84.3
**7.5**	98.7	96.7	75.7	75.7
**8**	89.2	98.7	66.2	77.7
**9**	80.7	90.4	57.7	69.4
**10**	74.2	89.6	51.2	68.6
**11**	63.1	73.5	40.1	52.5

Units: q_e_ in mg/g,(C_0_ = 20 mg/L, V = 0.10 L, m = 0.020 g; 25 °C).

### 3.3. Adsorption isotherms

Adsorption isotherms elucidate how Cu²⁺ or Zn² ⁺ interact with the modified bentonite surfaces and are essential for understanding mechanisms, designing treatment systems, and benchmarking performance. Equilibrium data for Cu²⁺ and Zn² ⁺ uptake on NWF and HWF modified bentonite were fitted to the Langmuir and Freundlich models, which respectively represent monolayer adsorption on energetically uniform sites and sorption on heterogeneous surfaces.

#### 3.3.1. Langmuir isotherm.

**Fig 5:**
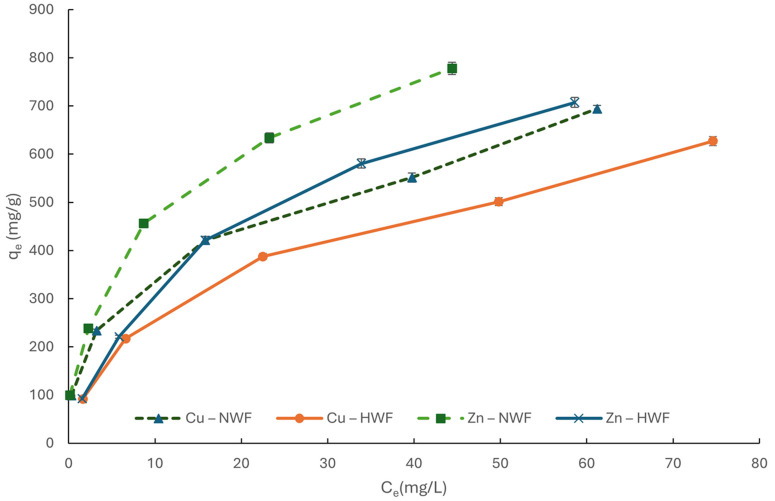
Langmuir isotherm data plotted as q_e_ vs C_e_. The solid lines represent the best fit of the nonlinear Langmuir model (Eq. 2) to the experimental data (points). Conditions: V = 0.1 L; m = 0.02 g; C_0_ = 20–200 mg/L; 25 °C.

The Langmuir model (Equation 2) assumes monolayer coverage on a finite number of identical sites with no lateral adsorbate interactions, and is written as:


$$\begin{array}{c} q_{e}=\frac{q_{m\;\;}K_{L}C_{e}}{\text{1}+K_{L}C_{e}} \end{array}$$ (2)

where *C*_*e*_ (mg/L) is the equilibrium concentration, *q*_*e*_ (mg/g) is the uptake at equilibrium, $q_{m\;\;}$(mg/g) is the monolayer capacity and *K*_*L*_ is the Langmuir constant. Nonlinear regression of *q*_*e*_ *versus C*_*e*_ provided excellent fits *(R*^*2*^_*adj*_*>0.99)* for all systems (Fig 5) and the fitted parameters are summarized in Table 2. Under the low-dose condition (m = 20 mg), NWF exhibited slightly higher *q*_*m*_ than HWF for both metal ions: *q*_*m*_ = 820 mg/g (Cu²⁺) and *q*_*m*_ = 905 mg/g (Zn²⁺) for NWF. In case of HWF, *q*_*m*_ = 760 mg/g for Cu^2+^, and *q*_*m*_ = 835 mg/g for Zn^2+^. These results indicate predominant monolayer uptake on accessible sites, consistent with enhanced site availability after acid activation and additional functional groups introduced by chitosan.

**Table 2:** Langmuir and Freundlich isotherm parameters for evaluating adsorption of Cu²⁺ and Zn²⁺ on NWF and HWF.

Metal ion	Adsorbent	q_max_ (mg/g)	K_L_ (L/mg)	R^2^_adj_ (Langmuir)	K_F_	1/n	n	R^2^_adj_ (Freundlich)
**Cu^2+^**	NWF	820	0.020	0.990	150	0.366	2.73	0.998
**Cu** ^**2+**^	HWF	760	0.018	0.988	77.2	0.569	1.76	0.976
**Zn** ^**2+**^	NWF	905	0.022	0.992	79.9	0.471	2.12	0.952
**Zn** ^**2+**^	HWF	835	0.020	0.990	195	0.353	2.83	0.980

#### 3.3.2. Freundlich isotherm.

**Fig 6:**
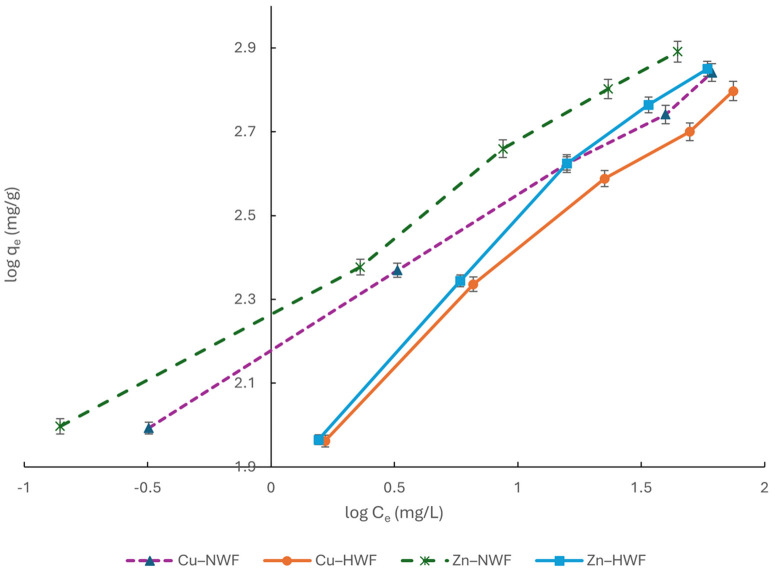
Freundlich linear plots (log q_e_ vs log C_e_) for Cu^2^^+^ and Zn^2^^+^ on NWF and HWF. The slope gives 1/n and the intercept gives log K_F_.

For comparison, the Freundlich model $q_{e}=KF{C_{e}}^{\left(\text{1}/n\right)}$ was applied for surface heterogeneity [12,32]. As shown in Fig 6, linearized plots (log *q*_*e*_ vs log *C*_*e*_) yielded high linearity (*R*^*2*^_*adj*_ ranged from 0.952–0.998). The *1/n* values ranged from 0.353 to 0.569 (n = 1.76–2.83), indicating favorable adsorption (0 < 1/n < 1) on heterogeneous surfaces. The corresponding *K*_*F*_ constants were 77–195(mg/g)(L/mg)(1/n), reflecting strong affinity under the tested conditions. While Freundlich captured heterogeneity well, yielding an excellent fit for Cu²⁺ on NWF (*R*^*2*^_*adj*_ = 0.998), the Langmuir model generally provided superior fits for the other three systems (*R*^*2*^_*adj*_ values of 0.988–0.992). This suggests that while heterogeneity is present, monolayer uptake on accessible sites remains a dominant mechanism under these conditions.

### 3.4. Adsorption kinetics

#### 3.4.1. First-order kinetic model.

The first-order kinetic model, $ln\left(q_{e}-\;q_{t}\right)=lnq_{e}-\;k_{\text{1}}t$, was evaluated for comparison. Adsorption capacities, *q*_*t*_ (mg/g), were obtained from Equation 3:


$$\begin{array}{c} q_{t}=\;\frac{V\times \left(C_{\text{0}}-C_{t}\right)}{m} \end{array}$$ (3)

Although linear fits could be constructed from early-time data, first-order model generally provided poorer agreement (R² ≈ 0.90–0.92) and *q*_*e*_ showed deviations. Therefore, this model is not considered the controlling kinetic expression in this study and detailed parameters are summarized in Table 3 [23,33].

**Table 3:** Kinetic parameters for Cu^2+^ and Zn^2+^ adsorption on NWF and HWF.

Metal ion	Sorbent	${𝐪}_{e}$ (exp)	${𝐪}_{𝐞}$ (PFO)	${𝐤}_{1}$ (min ⁻ ¹)	R^2^ (PFO)	${𝐪}_{e}$(PSO)	${𝐤}_{2}$ (g/mg·min)	R^2^ (PSO)
**Cu** ^**2+**^	NWF	0.93	0.75	0.047	0.915	0.91	0.088	0.998
**Cu** ^**2+**^	HWF	0.84	0.68	0.041	0.902	0.83	0.072	0.997
**Zn** ^**2+**^	NWF	0.82	0.69	0.045	0.910	0.81	0.084	0.997
**Zn** ^**2+**^	HWF	0.76	0.61	0.038	0.897	0.75	0.070	0.996

Units: *q*_*e*_ in mg/g; *k*_*1*_ in min^−1^; *k*_*2*_ in g·mg^−1^·min^−1^; *R²* dimensionless.

#### 3.4.2. Pseudo-second-order kinetic model.

**Fig 7:**
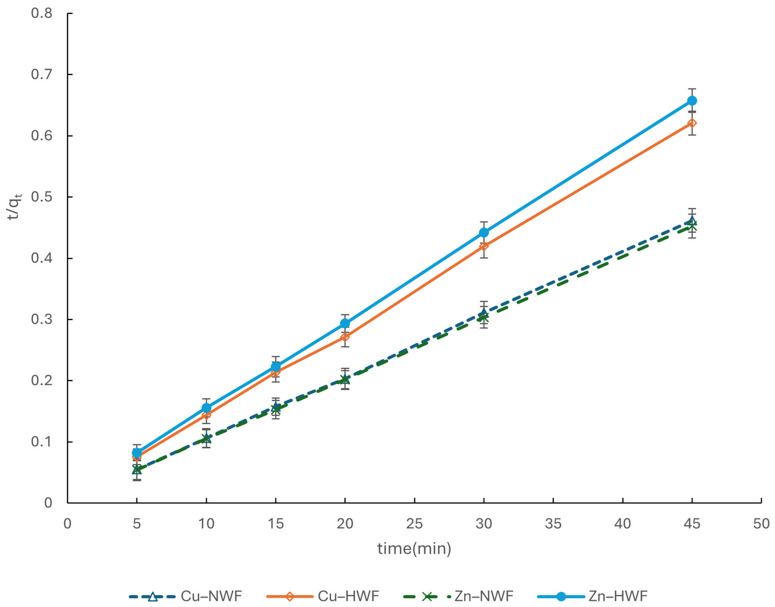
Pseudo-second-order plots: t/qt vs time for Cu²⁺ and Zn²⁺ on NWF and HWF; slopes and intercepts yield q_e_ and k_2_. Conditions: C_0_ = 20 mg/L; V = 0.10 L; m = 0.020 g; 25 °C.

The pseudo-second-order Kinetic model assumes chemisorption as the rate-limiting step and is expressed as Equation 4:


$$\begin{array}{c} \frac{t}{q_{t}}=\frac{\text{1}}{k_{\text{2}}q_{e}^{\text{2}}}+\frac{t}{q_{e}} \end{array}$$ (4)

where *k*_*2*_ (g·mg ⁻ ¹·min ⁻ ¹) is the rate constant, *q*_*e*_ (mg/g) is the theoretical equilibrium capacity, and $q_{t}$ (mg/g) is the adsorption capacity at contact time, t (min). Plots of *t/q*_*t*_ versus *time* (Fig 7) yielded highly linear relationships for all systems. NWF exhibited slightly higher apparent rates and faster approach to equilibrium than HWF, consistent with enhanced site accessibility after acid activation.

Time-dependent capacities (Fig 7) show a rapid uptake during the first 10–15 min of reaction, followed by a gradual approach to equilibrium after 20–30 min of reaction for both metal ions on both sorbents. Under the test conditions, NWF consistently reached equilibrium faster and attained slightly higher *q*_*t*_ than HWF. The superior pseudo-second-order fits (in comparision with first-order model), together with the time profiles, indicate that surface-controlled chemisorption—via ion exchange and surface complexation on accessible sites—dominates the overall rate. These kinetic findings are consistent with the isotherm analysis (monolayer coverage on accessible sites) as described in Langmuir section (3.3.1). Additionally, NWF exhibited higher apparent rates and reached equilibrium slightly faster than HWF for both metal ions, consistent with enhanced porosity and site accessibility after HNO₃ activation and calcination. Although HWF showed slightly lower initial uptake, its equilibrium capacities approached those of NWF, attributable to amine-bearing functional groups introduced by chitosan intercalation. Overall, acid-activated Suravajin bentonite, particularly the HNO₃-treated (NWF) form, provides fast and efficient sequestration of Cu²⁺ and Zn²⁺ under the tested conditions. The kinetic parameters from both models are summarized in Table 3 [33,34].

### 3.5. Effect of contact time

Contact time plays an important role on removal efficiency and the operative rate steps in adsorption. Here, Cu²⁺ and Zn² ⁺ uptake onto the two modified bentonites—NWF and HWF—was monitored under baseline conditions (*C*_*0*_ = 20 mg/L*; V* = 0.10 L*; m =* 20 mg). Residual concentrations were measured at different times as follows: *t* = 5, 10, 15, 20, 30,45 min. Adsorption capacities were calculated by Equation 5:


$$\begin{array}{c} q_{t}=\;\frac{V\times \left(C_{\text{0}}-C_{t}\right)}{m} \end{array}$$ (5)

As shown in Table 4, uptake increased sharply during the first 10–15 min for all systems, followed by a gradual approach to equilibrium. For both metal ions, equilibrium was effectively reached within 20–30 min. For NWF in case of Cu^2+^ adsorption, $q_{t}$ rose from 91.2 mg/g at 5 min to 98.7 mg/g at 20 min(*Δq*_*t*_ ≈ 7.5 mg/g), while on HWF, we see increase in $q_{t}$ from 66.2 to 73.7 mg/g over the same period (*Δq*_*t*_ ≈ 7.5 mg/g). A similar behavior was observed for Zn^2+^: *q*_*t*_(Zn) on NWF increased from 92.0 to 99.2 mg/g, whereas on HWF it showed higer values from 61.0 to 68.2 mg/g (*Δq*_*t*_ ≈ 7.2 mg/g). Despite comparable early-time gains, the equilibrium level on NWF remained around 25–31 mg/g higher than on HWF for both metal ions, indicating faster kinetics and higher attainable capacity on NWF.

**Table 4:** Time‑dependent adsorption capacity (q_t_) for Cu²⁺ and Zn²⁺ at C_0_ = 20 mg/L;V = 0.1 L; m = 0.020 g.

	*q*_*t*_ (mg/g)			
Time (min)	Cu^2 +^ –NWF	Cu^2 +^ –HWF	Zn^2 +^ –NWF	Zn^2 +^ –HWF
**5**	91.20	66.20	92.00	61.00
**10**	94.30	69.30	95.30	64.30
**15**	95.20	70.20	98.40	67.40
**20**	98.70	73.70	99.20	68.20
**30**	96.50	71.50	98.90	67.90
**45**	97.50	72.50	99.40	68.40

**Fig 8:**
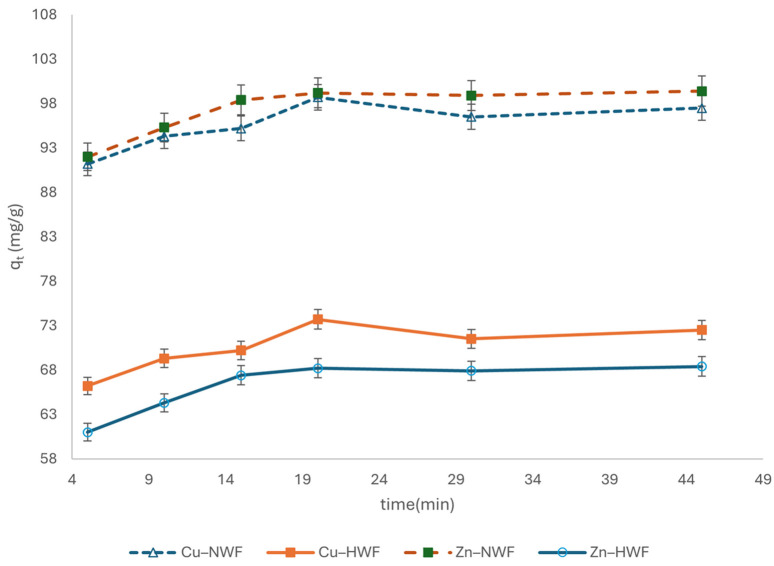
Adsorption capacity (q_t_ in mg/g) as a function of contact time (min) for Cu²⁺ and Zn²⁺ on NWF and HWF. Conditions: C_0_ = 20 mg/L; V = 0.10 L; m = 0.020 g; 25 °C.

As shown in Table 4 and Fig 8, the time related to ~95% of the equilibrium capacity (t_95_) was around 10–15 min for Cu^2+^ on NWF and 15–20 min on HWF, consistent with the larger apparent rates derived from the pseudo-second-order model (Fig 8; R² > 0.996). After 20 min, changes in *q*_*t*_ were higher than 2 mg/g for all cases (e.g., Cu/NWF: 98.7 → 97.5 mg/g; Zn/NWF: 99.2 → 99.4 mg/g), confirming that once equilibrium is approached, film/intraparticle diffusion effects become minor. Overall, these results indicate that under the tested conditions a contact time of 20–30 min is sufficient and highlight the role of surface chemistry and porosity: NWF benefits from enhanced site accessibility after HNO₃ activation and calcination, whereas HWF—despite a slightly slower approach—maintains stable capacities owing to amine-bearing functional groups introduced by chitosan intercalation. Table 4 clearly shows the progression of metal ion adsorption over time on each sorbent.

### 3.6 Effect of initial concentration of metal ions

**Fig 9:**
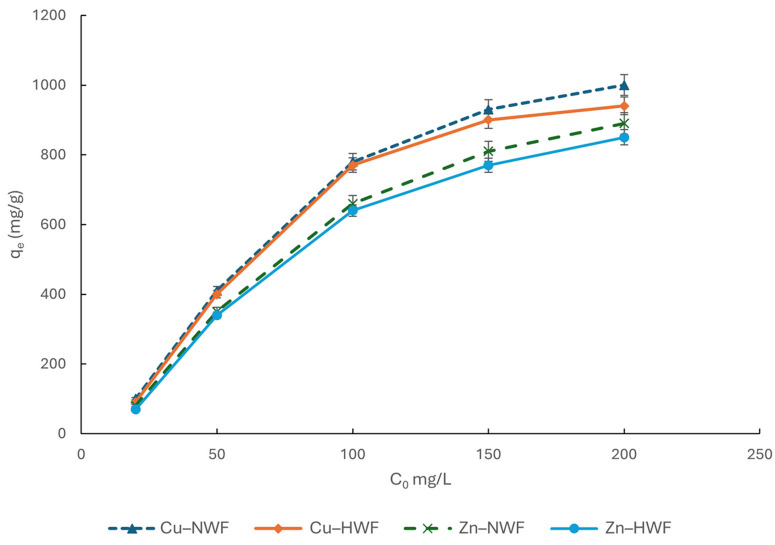
Effect of initial concentration (C_0_) on qe for Cu²⁺ and Zn²⁺ on NWF and HWF (C_0_ = 20–200 mg/L; V = 0.10 L; m = 0.020 g; 25 °C).

The initial metal ion concentration (*C*_*0*_) determines the driving force for mass transfer between the solution and the sorbent surface, thereby influencing the overall adsorption capacity. To evaluate this effect, batch adsorption tests were performed for Cu²⁺ and Zn² ⁺ . As shown in Fig 9 and Table 5, *q*_*e*_ represents a monotonically increase with *C*_*0*_ for both metal ions and both sorbents.This is attributed to the stronger concentration gradient and greater availability of metal ions to occupy active sites at higher *C*_*0*_. The rate of increase diminished at higher concentrations, consistent with progressive site saturation and increasing competition for a finite number of active centers on the bentonite surface. The slightly higher capacities on NWF across the entire range indicate that HNO₃ activation and subsequent calcination exposed more accessible sites and improved pore accessibility, in agreement with SEM/XRD observations. These trends are consistent with prior studies on modified clays reporting concentration-dependent enhancements in uptake until near-saturation is approached [3,8,13].

**Table 5:** Effect of initial metal ion concentration on adsorption capacity (qₑ) for Cu²⁺ and Zn² ⁺ .

	*q*_*e*_ (mg/g)			
C_0_ (mg/L)	Cu—NWF	Cu—HWF	Zn—NWF	Zn—HWF)
**20**	100	90	80	70
**50**	410	400	350	340
**100**	780	770	660	640
**150**	930	900	810	770
**200**	1000	940	890	850

### 3.7. Effect of sorbent dosage

**Fig 10:**
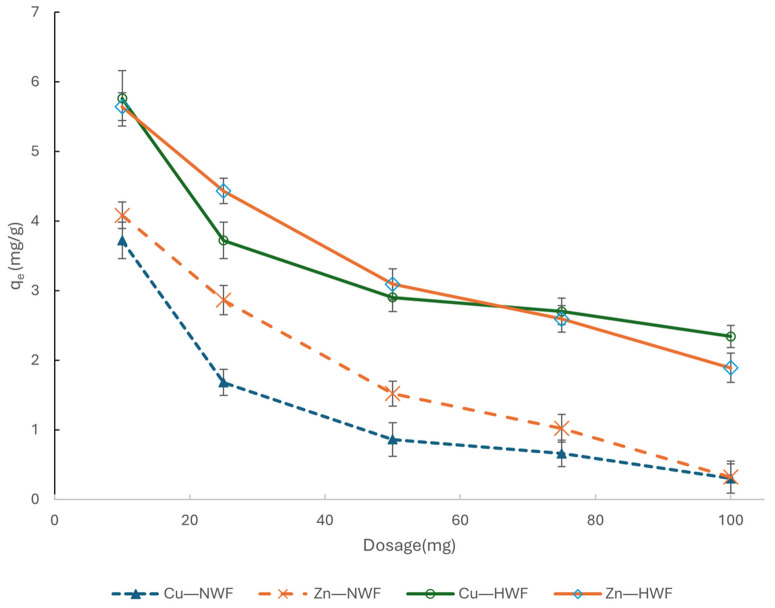
Effect of sorbent dosage on qe for Cu²⁺ and Zn²⁺ on NWF and HWF at C_0_ = 20 mg/L (V = 0.10 L).

Sorbent dosage affects both the removal percentage and the mass-normalized capacity (*q*_*e*_). To assess this effect, batch tests were performed at a fixed initial concentration (*C*_*0*_ = 20 mg/L) in 100 mL solution while varying the adsorbent mass from 10 to 150 mg. As summarized in Table 6 and illustrated in Fig 10, *q*_*e*_ decreases as the dosage increases for both metal ions and both sorbents This inverse trend is typically attributed to two main factors: (i) particle aggregation at higher solid loadings, which reduces the effective external surface area per unit mass, and (ii) an excess of available adsorption sites compared to the dissolved metal ions, resulting in incomplete site utilization and consequently lower mass-normalized capacities [3,32]. Notably, while *q*_*e*_ decreases with sorbent dosage increase, the removal percentage *(C*_*0*_ *− C*_*e*_*)/C*_*0*_ typically increases, underscoring the need to optimize dosage for both performance and cost.

**Table 6:** Effect of sorbent dosage on mass normalized capacity (q_e_) for Cu²⁺ and Zn² ⁺ .

Sorbent Dosage(mg)	*q*_*e*_(mg/g)			
	Cu—NWF	Zn—NWF	Cu—HWF	Zn—HWF
**10**	3.72	4.076	5.76	5.644
**25**	1.68	2.86	3.72	4.428
**50**	0.86	1.52	2.90	3.088
**75**	0.66	1.02	2.70	2.588
**100**	0.30	0.32	2.34	1.888

Conditins: *C*_*0*_ = 20 mg/L (*V* = 0.10 L; 25 °C),Units: Dosage in mg; *q*_*e*_ in mg/g.

At the lowest dosage (10 mg), *q*_*e*_ values were highest (e.g., Cu^2+^: 3.72 mg/g on NWF and 5.76 mg/g on HWF; Zn^2+^: 4.08 mg/g on NWF and 5.64 mg/g on HWF), and they show a progressive decline with increasing sorbent dosage. All reported capacities are consistent with the mass balance ceiling for each test (*q*_*e*_ and *q*_*max*_ = *C*_*0*_ *V/ m*), confirming internal consistency of the data. Data for dosages above 100 mg (e.g., 150 mg) were excluded from the analysis. At such high solid-to-liquid ratios (with a low *C*_*0*_), the*(C*_*0*_ *− C*_*e*_*)* term becomes extremely small and approaches the analytical uncertainty of the measurement, leading to unreliable *q*_*e*_ values, as highlighted in methodological best-practice reviews [32].

The relative behavior is consistent with earlier sections: NWF benefits from improved site accessibility after HNO_3_ activation and calcination, whereas HWF shows comparatively high capacities at low dosage due to amine-bearing chitosan functionalities.

### 3.8. Thermodynamic of adsorption

To understand the spontaneity and thermal nature of the adsorption, a thermodynamic analysis was performed at 20, 30, and 45 °C. The key thermodynamic parameters (Equation 6,7)—standard Gibbs free energy (*ΔG°*), enthalpy (*ΔH°*), and entropy (*ΔS°*)—were determined from the temperature dependence of the distribution coefficient (*K*_*c*_ *= q*_*e*_*/C*_*e*_) using the classic Van’t Hoff equations:


$$\begin{array}{c} nK_{c}=-\Delta H\circ /(RT)\;+\;\Delta S\circ /R\; \end{array}$$ (6)

$$\begin{array}{c} \Delta G\circ \;=\;-RT\;ln\;K_{c} \end{array}$$ (7)

with R = 8.314 J·mol ⁻ ¹·K ⁻ ¹ and T (K) the absolute temperature. The calculated parameters for all systems are summarized in Table 7. These results provide clear insights into the adsorption mechanism. For all systems, the consistently negative *ΔG°* values confirm the spontaneous nature of the adsorption process. The positive *ΔH°* values reveal that the uptake is endothermic, indicating that higher temperatures favor the adsorption.

**Table 7:** Thermodynamic parameters from Van’t Hoff analysis.

Metal ion	Sorbent	ΔH°	ΔS°	-ΔG° (20 °C)	ΔG° (30 °C)-	-ΔG° (45 °C)	R²
**Cu** ^**2+**^	NWF	14.6	81.7	9.77	9.33	13.10	0.99
**Cu** ^**2+**^	HWF	4.9	45.8	8.48	7.80	9.62	0.99
**Zn** ^**2+**^	NWF	9.2	67.0	11.53	10.26	11.43	0.99
**Zn** ^**2+**^	HWF	9.1	59.7	9.35	8.70	9.12	0.98

*Units:* Δ*H° (kJ·mol ⁻ ¹);* Δ*S° (J·mol ⁻ ¹·K ⁻ ¹);* Δ*G° (kJ·mol ⁻ ¹); R² (dimensionless)*.

Furthermore, the positive *ΔS°* values suggest an increase in randomness at the solid-liquid interface during adsorption. This is a common finding and is attributed to the release of structured water molecules from the hydrated metal ions as they adsorb onto the bentonite surface. This displacement of water molecules increases the overall entropy of the system [35].

Thermodynamic parameters obtained from the Van’t Hoff relation showed consistent signs and trends across all systems, confirming the feasibility and spontaneity of the adsorption process.. Notably, the NWF material exhibited slightly more favorable thermodynamics (i.e., more negative *ΔG°* and higher *ΔS°*) than HWF, which aligns well with its faster kinetics and higher adsorption capacities observed in the preceding sections.

## 4. Conclusion

This study demonstrated that Iranian bentonite obtained from the Suravajin Aghigh mine, when chemically modified as NWF (HNO_3_-activated and calcined) and HWF (HCl-activated and chitosan-intercalated), could yield an effective sorbent for removing Cu^2+^ and Zn^2+^ from water. Equilibrium data were best described by the Langmuir isotherm (*R*^*2*^_*adj*_ > 0.98), indicating predominant monolayer adsorption on accessible sites. Kinetic analysis showed excellent agreement with the pseudo-second-order model (R^2^ > 0.996), confirming a chemisorption-controlled process. Thermodynamic results evidenced spontaneous, endothermic uptake with increased interfacial disorder, and higher temperatures favored adsorption. Material characterization (XRD, FTIR, SEM) verified successful acid activation/chitosan intercalation and revealed rougher, more porous surfaces—particularly for NWF—consistent with enhanced site accessibility. Furthermore, a critical analysis of the isotherm data (Table 5) indicates that the adsorbent did not reach saturation under the tested concentration range. The experimentally measured *q*_*e*_ for Cu-NWF (1000 mg/g) exceeded the model-extrapolated *q*_*m*_ value (820 mg/g), suggesting the true maximum capacity may be even higher. Therefore, the most scientifically rigorous benchmark is the demonstrated experimental capacity (*q*_*e*_). This demonstrated *q*_*e*_ of 1000 mg/g is highly competitive and significantly exceeds the maximum theoretical capacities (*q*_*m*_) reported for many other modified bentonite and clay-based adsorbents, which often range from 20–150 mg/g [11,36,37]. Given its local availability, low cost, and robust performance under practical conditions, NWF and HWF modified Iranian (Suravajin) bentonite is a strong, sustainable candidate for real-world wastewater treatment applications targeting Cu^2+^ and Zn^2+^ in heavy metal–impacted sectors. While this study demonstrates high adsorption capacities, further investigations are needed to evaluate the performance of NWF and HWF in real industrial wastewater and their regeneration potential.

## Supporting information

S1 Data Raw experimental data for Cu(II) and Zn(II) adsorption experiments, including adsorption capacity, isotherm, kinetic, and thermodynamic datasets used in this study. (RAR)
